# Differential Effects of Dietary Supplementation of Krill Meal, Soybean Meal, Butyrate, and Bactocell^®^ on the Gene Expression of Atlantic Salmon Head Kidney

**DOI:** 10.3390/ijms21030886

**Published:** 2020-01-30

**Authors:** Mahsa Jalili, Marco Gerdol, Samuele Greco, Alberto Pallavicini, Francesco Buonocore, Giuseppe Scapigliati, Simona Picchietti, Maria Angeles Esteban, Morten Rye, Atle Bones

**Affiliations:** 1 Cell, Molecular Biology and Genomics Group, Department of Biology, Faculty of Natural Sciences, Norwegian University of Science and Technology, NO-7034 Trondheim, Norway; 2 Department of Life Sciences, University of Trieste, 34100 Trieste, Italy; 3 Department for Innovation in Biological, Agro-food and Forest systems (DIBAF), University of Tuscia, 01100 Viterbo, Italy; 4 Cell Biology and Histology Department, Faculty of Biology, University of Murcia, 30100 Murcia, Spain; aesteban@um.es; 5 BioCore, Department of Clinical and Molecular Medicine, NTNU―Norwegian University of Science and Technology, NO-7491 Trondheim, Norway

**Keywords:** innate immunity, complement system, diet, fish, transcriptomics, RNA-seq

## Abstract

The head kidney is a key organ that plays a fundamental role in the regulation of the fish immune response and in the maintenance of endocrine homeostasis. Previous studies indicate that the supplementation of exogenous dietary components, such as krill meal (KM), soybean meal (SM), Bactocell^®^ (BA), and butyrate (BU), can have a significant effect on the immune function of the head kidney. The aim of this study was to investigate the differential effect of these four dietary ingredients on the transcriptional profiles of the head kidney of the Atlantic salmon. This study revealed that just a small number of genes were responsive to the feeding regime after a long-term (12 weeks) treatment, and evidenced that the most significant alterations, both in terms of the number of affected genes and magnitude of changes in gene expression, were detectable in the BU- and KM-fed groups compared with controls, while the SM diet had a nearly negligible effect, and BA had no significant effects at all. Most of the differentially expressed genes were involved in the immune response and, in line with data previously obtained from pyloric caeca, major components of the complement system were significantly affected. These alterations were accompanied by an increase in the density of melanomacrophage centers in the KM- and SM-fed group and their reduction in the BU-fed group. While three types of dietary supplements (BU, KM, and SM) were able to produce a significant modulation of some molecular players of the immune system, the butyrate-rich diet was revealed as the one with the most relevant immune-stimulating properties in the head kidney. These preliminary results suggest that further investigations should be aimed towards the elucidation of the potential beneficial effects of butyrate and krill meal supplementation on farmed salmon health and growth performance.

## 1. Introduction

The head kidney can most certainly be considered as one of the most important immune organs in teleosts, due to the massive presence of lymphocyte and macrophage populations able to mount a strong and efficient immune response [1]. Due to its fundamental role in fish immunity, the head kidney may be an important target tissue for elucidating the immunomodulatory effects of dietary supplements, which may find a practical application in fish aquaculture through the improvement of resistance towards pathogenic infections, fish growth, and marketability of the final product [2]. However, the studies carried out so far on this subject have mostly investigated systemic immune parameters, the histological alterations and inflammatory processes occurring in the digestive system, neglecting the modulation of gene expression in this important immune tissue.

The fish head kidney can also be considered as an organ analogous to the mammalian adrenal gland, as it produces and secretes several types of hormones, such as cortisol, catecholamines, growth hormone, prolactin, melanin-concentrating hormone, proopiomelanocortin, and others. Due to the contemporary presence of these endocrine hormone-producing cells and cytokine-producing lymphoid cells, the teleost head kidney emerges as one of the key players in the regulation of the immune-neuro-endocrine circuitry, through the reciprocal signaling between the endocrine and immune systems. In particular, the pro-inflammatory cytokines produced by immune cells might exert a negative paracrine modulatory activity on the thyroid follicular cells present in the head kidney [3,4,5].

Multiple lines of evidence support the anti-inflammatory properties of dietary supplements based on Antarctic krill (*Euphausia superba*) in human and experimental animal models [6,7]. Several studies have similarly suggested that the nutritional properties of krill may provide significant benefits to the health of farmed fish species [8,9,10]. For example, an increased dietary intake of chitin, one of the main components of krill, potentiates phagocytosis, complement system activity, and respiratory burst, leading an increased protection against aquatic bacterial infection [11,12,13]. Unfortunately, the digestibility of chitin is very low in salmon, to the point that a high dose meal (>60% krill) may have detrimental effects on fish growth rates, leading to the development of irregular vacuoles in the enterocytes of the distal intestine [14]. Hence, the use of small-size chitin chains, which maintain their immune-stimulant properties, would be preferable [15]. Krill meal also contains high doses of β-carotene, whose antioxidant properties can lead to activation of the alternative pathway of the complement system, also contributing to an increase of the phagocytic activity in teleost species [16]. Moreover, feeds with high protein content, such as krill meal, have the potential to further enhance immune system performance and resistance to disease [17,18].

Other dietary supplements largely used in fish aquaculture, based on soybean derivatives, have a well-established pro-inflammatory action on the salmon digestive system [19,20]. Soybean can influence the intestinal immune response by altering cell proliferation, apoptosis, and epithelial integrity, resulting in an increased exposure to antigens, consequently leading to a higher susceptibility to infections [21]. Experimental evidence further suggests that soybean meal supplementation can lead to the infiltration of lymphocytes, macrophages, and polymorphonuclear leukocytes in the lamina propria, paired with a marked increase in the number of eosinophilic granule cells in the luminal parts of the connective tissue [19]. Soybean meal may also influence the systemic levels of immunoglobulins, oxidative radicals, and circulating macrophages [22]. From a molecular perspective, such changes are accompanied by an increased expression of major histocompatibility complex class II, lysozyme activity, and immunoglobulin M in the brush border of intestinal cells [23].

The supplementation of probiotics in fish aquaculture has the potential to stimulate the immune response, due to the exposure of intestinal mucosa to highly immunogenic surface proteins that can be recognized as microbe-associated molecular patterns [24]. Probiotic supplementation can be also applied with success to the Atlantic salmon, as revealed by the upregulation of some important immune genes upon the treatment with probiotics derived from the Atlantic cod [25]. The immune-modulatory potential of one of the most widely used bacteria in this context, *Pediococcus acidilactici* (available in the commercial preparation Bactocell^®^), has been investigated in detail in *Nile tilapia*, leading to contrasting results [26,27,28]. Experiments carried out in other fish species seem to be concordant in reporting significant positive modulatory effects on the activity of lysozyme and the complement system, paired with a general improvement of cellular and humoral immune parameters [29,30].

Prebiotics have been frequently used as an environment-friendly alternative to antibiotics in fish aquaculture due to their relevant potential as immunostimulants [29]. Microbial fermentation metabolites derived from intestinal microbiota may indeed support the growth of other probiotic bacteria; moreover, these metabolites can also be taken up by epithelial intestinal cells, serving as a potential source of energy [31]. As a short chain fatty acid, one of these fermentation products, i.e., butyrate, can interact with G-protein-coupled receptors found on the surface of enteric epithelial cells and leukocytes, with significant effects on immune system function [32]. The experimental studies carried out so far in farmed fish species have led to controversial results on growth rate, overall health conditions and modulation of immune system markers [33,34], and further investigations will be required to clarify whether butyrate supplementation has a significant effect on fish innate and adaptive immunity.

Alterations of immune parameters may also be linked with a significant ultrastructural re-organization of the head kidney tissue. This has been described as being highly reminiscent of mammalian bone marrow, due to the presence of a reticular network of stromal cells (macrophage-like and fibroblast-like cells), which support different types of hematopoietic cells, as well as of sinusoidal endothelial cells associated with the thin vessels that provide the main blood supply to this tissue [35,36,37]. In this site, circulating exogenous pathogen-derived molecules present in the bloodstream may be taken up through receptor-mediated endocytosis [38], and free macrophages eliminate invading microbes through phagocytosis, bridging the two arms of innate and adaptive immunity by acting as antigen-processing cells [1]. Melanomacrophages, i.e., large pigmented dark cells found in the peripheral margin of the head kidney, are usually organized in melanomacrophage centers (MMCs) [39], and they are thought to play both non-immunological (e.g., the disposal of decayed hematopoietic cells [35]) and immunological functions, possibly linked with the establishment of immune memory [40] (e.g., they act as sites of antigen retention [41]).

Previous experiments have demonstrated that dietary supplementation has a moderate impact on the modulation of the expression of some immune-related genes in the pyloric caeca of Atlantic salmon at the fry stage [42]. Taking full advantage of the high-quality genome available for this species, the RNA sequencing approach used in this study allowed these observations to be extended to the head kidney, evaluating the effects of an 84 day-long supplementation of KM-, SM-, BA-, and BU-rich feeds. Although salmons can live up to eight years, this can be considered as a long-term experiment in the context of the fry developmental stage, since several morphological and physiological changes occur within a few weeks in preparation for the following life stages. The effects of the diets on the transcriptional profile were investigated, with a particular focus on markers related to the immune response, and some preliminary histological observations were also made on the modification of the head kidney tissue.

## 2. Results and Discussion

### 2.1. An Overview of the Effects of Different Feeds on Immune-Related Gene Expression in Head Kidney

The statistical analysis of differential gene expression revealed that just a very small number of genes underwent significant expression shifts in response to the four experimental diets, compared with the control group. In detail, a total of six differentially expressed genes (DEGs) were identified exclusively in response to the BU-rich diet, two DEGs in response to the KM-rich diet, and one DEG was shared by the two experimental groups. The single gene responsive to the SM-rich diet was also significantly modulated in response to BU (which essentially had the same ingredients of SM, supplemented with butyrate). No significant alteration of the gene expression levels could be detected in salmons fed with the BA-rich meal.

Overall, the 10 DEGs were: Vitellogenin (VTG), complement C3 (C3), microfibrillar-associated protein 4 (MFAP4), cerebellin-2-like protein (CBLN2L), complement factor H-like (CFHL), transmembrane 4L6 family member 4-like (TM4SF4), cadherin 4 (CDH4), apolipoprotein B-100-like (APOBL), complement factor H-related protein 5-like (CFHR5L), and complement factor B-like (CFBL). The DEG fold change (FC) values and statistical significance of the differential expression are reported in Table 1.

**Figure 1:**
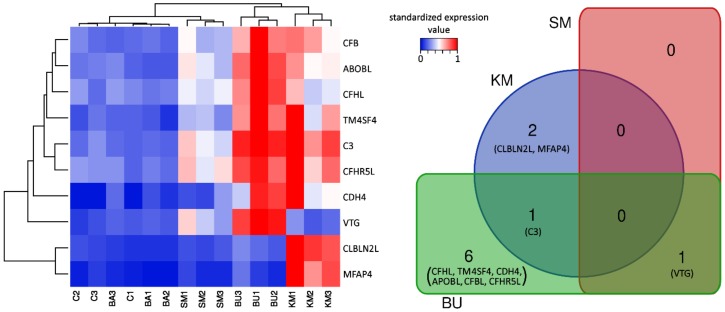
Heat map summarizing the expression levels of the 10 DEGs identified across the 5 experimental groups, and the overlap between differentially expressed genes in response to the diets. C: control diet; KM: krill meal-rich diet; SM: soybean meal-rich diet; BA: Bactocell^®^-rich diet; BU: butyrate-rich diet. See the main text for the acronyms of the gene names. Hierarchical clustering, based on the Euclidean distance and average linkage, was applied to genes and samples, which included three biological replicates. Clustering was based on the TPM expression values, standardized on the sample achieving the highest expression levels. Dendrograms close to the X and Y axes show the similarity of the expression trends among samples and genes, respectively. DEGs: Differential Gene Expression; TPM: Transcripts Per Million.

In general, the comparison of the gene expression profiles among the five experimental groups clearly showed a close match between C and BA, whose biological replicates were intermixed in the heat map (Figure 1). In addition, sample clustering revealed a common signature of transcriptional activation in the KM and BU groups, even though the two groups showed some peculiarities and only shared a single DEG. Indeed, while most of the other DEGs identified in BU visibly displayed a similar expression trend in KM, most of them did not reach the threshold for statistical significance, most likely due to the higher variability of the gene expression profiles observed in the three biological replicates (Figure 1). On the other hand, the SM diet only produced very minor changes and was consequently clustered close to C and BA, with a single DEG identified.

While the low number of DEGs identified in this study did not allow pathway enrichment analysis to be carried out to identify significantly altered biological pathways, the majority of the aforementioned DEGs were well-known molecular players of the complement system, or had been previously linked with immune-related functions, either in Atlantic salmon or in other teleost fish species. The possible implications of the DEGs identified in this study in the functional modulation of the head kidney in response to long-term exposure to different dietary ingredients will be discussed in detail in the following sections, with a particular focus on their involvement in the immune response.

### 2.2. Validation of RNA-Seq Data by RT-qPCR

**Figure 2:**
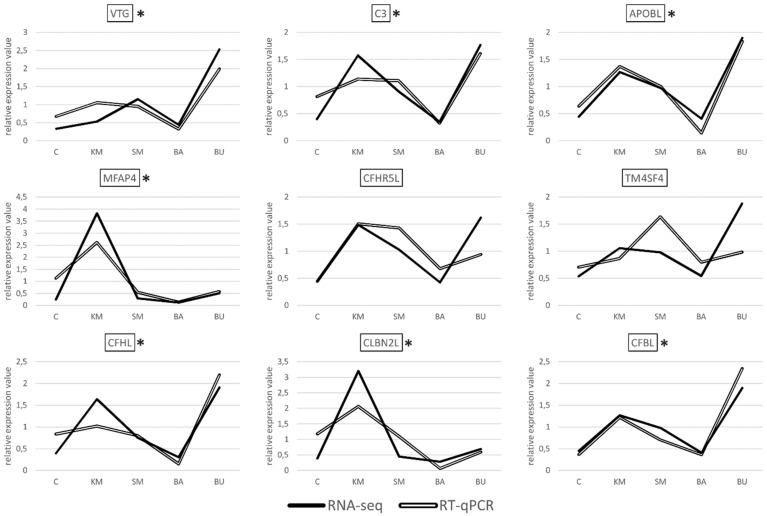
Pairwise comparison between the gene expression trends observed in RNA-seq and RT-qPCR analyses, for the nine selected DEGs. In order to enable an easier interpretation of the expression data, the gene expression values obtained for RNA-seq (TPM) and RT-qPCR (expression values relative to the two housekeeping genes used for internal normalization) were normalized on the mean value calculated across the five experimental samples. C: control; KM: krill meal; SM: soy meal; BA: Bactocell^®^; BU butyrate. Target genes displaying a statistically significant correlation between the gene expression trends observed for the two species are marked by an asterisk.

Albeit with different degrees of overlap, the gene expression datasets obtained with RT-qPCR showed a highly significant correlation with the RNA-seq results for seven out of the nine transcripts selected for validation, i.e., VTG, MFAP4, CFHL, CFBL, APOBL, C3, and CBLN2L, (Figure 2), with a R^2^ Pearson correlation coefficient equal to 0.7498, and a *p*-value < 0.00001.

In stark contrast with these results, the correlation was not significant for the two remaining targets, i.e., CFHR5L and TM4SF4. These discrepancies are most likely due to technical factors linked to the different methodologies used for quantification. Namely, RNA-seq makes use of all the reads matching to an annotated genomic feature (in this case, the target genes), regardless of their specific mapping coordinates. This implies that reads originated from all the possible splicing variants are taken into account to calculate a cumulative gene expression value for a given gene. On the other hand, RT-qPCR assays are based on the design of primers aimed at the amplification of relatively short amplicons, and therefore it targets only a very small portion of the selected transcripts. Consequently, RT-qPCR can only provide quantitative data concerning the splicing isoforms matched by the primers. The PCR efficiency might also differ across samples and targets, either due to the presence of single nucleotide polymorphisms (SNPs), differences in the primer melting temperature, or the amplicon size. Altogether, these factors can explain the apparent lack of a correlation, which is frequently observed in studies that compare the outcomes of RNA-seq and PCR-based approaches [43].

Overall, the experimental validation of the gene expression trends of seven out of the nine selected genes by RT-qPCR confirms the reliability of our approach and the appropriateness of the significance thresholds used for the detection of differential gene expression.

### 2.3. Modulation of Complement Proteins in Response to BU- and KM-Rich Diets

In bony fish species, the complement system plays a pivotal role at the crossroads between the innate and adaptive immune response. Some important components (such as C3 and CFBL) have undergone gene duplication along teleost evolution, enabling an expansion of innate immune functions, which might provide an improved modulation of adaptive immunity through the binding to cell-surface receptors found in dendritic cells and lymphocytes [44]. The complement system can be schematically divided into three main “arms”, i.e., (i) the lectin pathway, which involves lectins (e.g., mannose-binding lectins and ficolins), carbohydrate-binding proteins that can bind to the surface of pathogens to induce opsonization; (ii) the classical pathway, which is activated upon the interaction between the C1q complex and antigen-complexed immunoglobulins; and (iii) the alternative pathway, which is activated in response to the direct binding of pathogens by C3 [44,45].

The three complement pathways, which share C3 as their central component, converge on the activation of downstream molecules through the activity of serine proteases that results in the production of anaphylactic and chemotactic components [46], in the modulation of an acquired immune response [47], and in the final activation of the membrane attack complex and lysis of invading cells [46,47]. Although most of the components of the mammalian complement system are synthesized in the liver, experimental evidence suggests that in bony fish, complement factors can also be produced in other organs, such as the muscle, heart, intestine, and head kidney [47]. Studies carried out in the Atlantic cod, halibut, and spotted wolffish have revealed that, in addition to its immune functions, the fish complement system may also play an important role in other processes, such as organ regeneration and development [46,47]. In line with these data, we recently reported that a relevant number of complement-related genes could be significantly modulated in the pyloric caeca of juvenile Atlantic salmon in response to first feeding [42].

C3, CFBL, CFHL, and CFHR5L, four core components of the complement system, were significantly upregulated in response to the BU-rich diet. Among these, only C3 significantly increased its expression in response to KM supplementation (Table 1). Although the detailed inspection of the gene expression profiles of CFBL, CFHL, and CFHR5L in individual salmons showed that these three complement-related genes followed a very similar expression trend (Figure 1), their increase of expression was not considered as statistically significant due to the high degree of inter-individual variation in the response among the three biological replicates.

Taking into account the role of the complement system in balancing the innate and adaptive immune response and in regulating normal inflammatory reactions [48], the upregulation of C3 in response to BU and KM feeding was a finding with important implications. In particular, C3 was the second most significantly overexpressed gene in BU, reaching expression values slightly lower than 1000 TPM, with logFC = 2.08, (see Figure 1 and Table 1). The simultaneous upregulation of the complement factor B (CFBL, LogFC = 1.81) also points towards an activation of the complement system, as this component is a fundamental driver of the activation of the proteolytic cascade of the alternative pathway: Upon the cleavage of factor B by factor D, the catalytic subunit Bb associates with the C3b fragment, forming C3 convertase. Nevertheless, the expression of the downstream complement component C5, post-translationally cleaved in C5a (initiator of the inflammation pathway) and C5b (initiator of the lytic pathway) [49], was not modulated by the diet.

Two other key complement system genes, CFHL and CFHR5L, showed an expression trend nearly identical to C3, albeit with much lower expression levels (<100 TPM, Figure 1). CFHL is a suppressor of the alternative pathway of the complement system, which acts by inhibiting the activation of C3b and C5b convertases [50]. CFHR5L is a member of the factor H protein family, which is found in human kidney glomeruli, where it colocalizes and binds to C3, contributing to the deposition of glomerular complement deposits [51]. CFHR5L may also act as a cofactor in accelerating the decay of C3 convertase [52], and studies carried out in a rat experimental model showed that its upregulation in the epithelial cells of glomeruli is associated with high levels of activity of the complement system, and in particular of the membrane attack complex [53]. Based on the known functions of CFHL and CFHR5L, the upregulation of these two genes may be interpreted as a response enacted to prevent the overactivation of the complement system, which would have obvious negative effects due to the inflammation-mediated tissue damage [54].

In addition to their role in complement activation and regulation, CFHR5L and other factor H-related proteins (FHRs) are associated with high-density plasma lipoprotein particles in human blood, where they may cover an accessory lipid transportation function [52,55]. The association of FHRs with lipoproteins may, to some extent, also explain the upregulation of APOBL in the BU group, a major component of low- and very low-density lipoparticles (this gene reached ~500 TPM, with LogFC = 1.99, see Figure 1 and Table 1).

### 2.4. Vitellogenin Expression was Strongly Induced by the BU-Rich Diet

VTG is a multifunctional pleiotropic reproductive and immuno-modulator phosphoglycoprotein, which is mainly produced by the liver in adult fish under strict estrogen control, released in the bloodstream, and finally used as the main constituent of egg yolk. VTG was the most significantly upregulated gene in the head kidney of the BU-fed group, as well as the only DEG identified in the SM-fed group. In BU, VTG showed a particularly strong increase in expression, reaching values as high as ~500 TPM, with LogFC = 2.83, while its induction in SM was moderate (see Figure 1 and Table 1). On the other hand, VTG did not undergo relevant modulation in KM- and BA- fed salmons. Previous reports have clearly demonstrated that estrogen and estrogen-like components can regulate the functioning of the immune system in teleost fish species [56]. Considering the mutual cross-talk between the endocrine and immune systems, and the key function of the head kidney in this context, an alteration of the transcriptional levels of estrogen-related factors often coincides with immune modulation [57].

In addition to its fundamental role as a nutrient resource in early larval development, VTG can also act as a fast responsive inducible pattern recognition receptor, with opsonin-like properties [58]. VTG is able to recognize non-self components of bacterial origin [59,60], inducing phagocytosis in salmon macrophages, possibly through the interaction with specific receptors present on the surface of these fish immune cells [59,61,62]. VTG has also been demonstrated to possess antiviral properties against salmonid infectious pancreatic necrosis virus, leading to the neutralization of the pathogen by its coating and aggregation [61].

To the best of our knowledge, the strong activation of VTG transcription in the BU-fed group does not find a clear justification in the existing scientific literature and, while its induction might be connected with the activation of the complement system, no evidence of a common regulatory network including VTG, C3, and CFBL has ever been reported to date. Further investigations should be directed at exploring whether this significant modulation can be explained within the frame of the previously reported immune-stimulating properties of butyrate [63]. On the other hand, the moderate upregulation of VTG in response to the SM feeding could also be tentatively linked with an induction of its secretion by phytoestrogens, since these molecules can mimic the action of the natural endogenous inducer of VTG production, 17-β estradiol [64].

### 2.5. Involvement of Microfibril-Associated Protein 4 in the KM-Rich Diet Response

We observed a highly significant increase in the gene expression of MFAP4 in the KM-fed group, as this gene peaked at ~500 TPM, compared with an expression level lower than 100 TPM in the other four experimental groups (see Figure 1 and Table 1). In teleost fish, which lack ficolins, MFAP4 can act as an alternative pathogen recognition receptor in the lectin pathway of the complement system [65]. Indeed, MFAP4 has been shown to act in collaboration with mannose-binding lectins in the recognition of carbohydrate moieties associated with pathogens, leading to their opsonization and killing though the activation of the lytic pathway of the complement system [66,67]. Although the precise mode of action of MFAP4 has not been fully elucidated yet, this protein is capable of binding to the collagen domain of surfactant protein D, and it structural organization displays the presence of a C-terminal fibrinogen-like domain (as in ficolins), paired with an integrin-binding domain in the N-terminal region, which confer cell adhesion properties to this protein [65,68].

The activation of MFAP4 in response to KM is consistent with the previously reported immuno-stimulant and complement-activating properties of chitin, whose content in KM feed was relatively high [12,69,70]. We might speculate that the overexpression of MFAP4 was triggered in response to the specific recognition of chitin components present in the KM-rich feed, recognized by unknown pattern recognition receptors as potential pathogen-associated molecular patterns. However, in the absence of an actual pathogen, this response was likely unable to significantly potentiate the activation of the entire complement system machinery, even though, as previously mentioned, some complement components displayed a visible, but not statistically significant, induction (Figure 1).

### 2.6. Possible Involvement of the Other DEGs and Immune Response

There are only limited reports about the function and expression patterns of the three remaining DEGs identified in this study in teleost fish species, i.e., TM4SF4, CDH4, and CBLN2L.

The expression of CBLN2L was exclusively induced in the KM-fed group (LogFC = 2.81, see Figure 1 and Table 1). This gene encodes a C1q domain-containing protein, which pertains to a relatively large family that takes its name from the three main chains of the complement C1q complex. Although human cerebellin is thought to act as synaptic organizers and as a neuromodulator, the rainbow trout orthologue has been shown to play a key role as an acute phase protein during the earlier phases of inflammation, in response to *Vibrio* sp., *Yersinia ruckeri*, and lipopolysaccharide (LPS) challenges, as well as in response to live vaccination with *Ichthyophthirius multifiliis* [71,72,73,74]. Other studies have further identified cerebellins in suppression subtractive hybridization libraries resulting from tissues (including the head kidney) collected from Atlantic salmon infected with *Aeromonas salmonicida* [75], and in expressed sequence tag libraries obtained from halibut vaccinated with *V. anguillarum* and *A. salmonicida* [76]. While these data point towards an immune function for fish CBLN2L, the possible connection between the upregulation of this gene and the activation of the complement system remains to be established.

TM4SF4 encodes a transmembrane protein of the tetraspanin family, which, in humans, is mainly thought to play a role in cell growth regulation [77]. This gene underwent a significant upregulation in the BU group, reaching 120–160 TPM (LogFC = 1.7, see Figure 1 and Table 1). TM4SF4 has been observed among the genes downregulated in response to acute infection with the viral hemorrhagic septicemia virus in the Pacific herring [78] and, in another study carried out in the Japanese flounder, it was modulated in various tissues (i.e., liver, head kidney, gill, and muscle) in response to *Vibrio anguillarum* challenges [79]. These two reports only provide indirect evidence of a possible involvement in the immune response and do not presently allow the formulation of a more comprehensive hypothesis concerning the functional link between TM4SF4 and the other genes involved in the molecular network of the DEGs activated by the BU-rich diet.

Although CDH4 was identified as a significantly upregulated gene in BU (LogFC = 4.17, see Figure 1 and Table 1), the very high FC value observed was due to the very low expression levels of this gene in the control group (i.e., <1 TPM). Members of the cadherin family act as adhesive components in barrier junctions and can regulate immune responses in dendritic cells, as well as in epithelial cells [80]. In the spleen of common carp, the gene expression of intercellular adhesion molecules, such as epithelial cadherin, were markedly affected in response to *Aeromonas hydrophila* experimental challenges. Although this protein is of high interest due to its barrier function, which can prevent uncontrolled passages of non-self components and ions via cellular pores and junctions [81], the extremely low expression levels observed in our study suggest that this type of cadherin is unlikely to play a relevant role in salmon head kidney.

### 2.7. Histological Analyses

**Figure 3:**
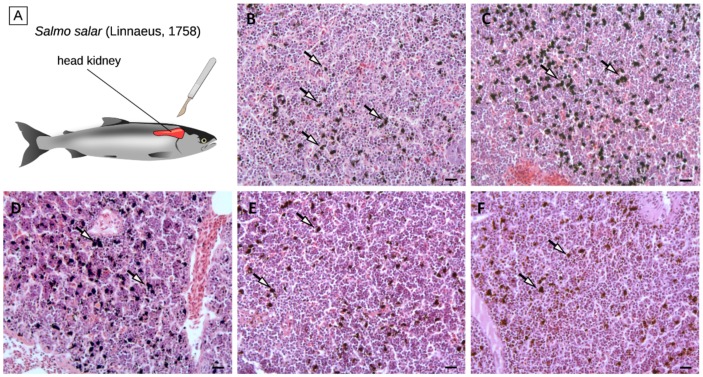
Schematic anatomy of Atlantic salmon, showing the location of the head kidney (panel **A**). Histological section of the head kidney from salmons fed with the control (panel **B**), krill meal (panel **C**), soybean meal (panel **D**), Bactocell^®^ (panel **E**), and butyrate-rich diets (panel **F**). Arrows indicate MMCs. Scale bar = 20 µm. MMCs: Melanomacrophages.

Despite the small number of DEGs identified in the comparison among the five diets, histological alterations could be observed in the head kidney tissue of the KM-, SM-, and BU-fed groups, and these changes could be mainly linked with a variation in the number and organization of melanomacrophage centers (Figure 3). In detail, histological analyses revealed that, while the density of MMCs was not affected by BA (103 ± 38 MMCs/100,000 µm^2^), it increased by ~20% in KM- and SM-fed salmon (147 ± 79 MMCs/100,000 µm^2^ and 148 ± 46MMCs/100,000 µm^2^, respectively) compared with controls (119 ± 63 MMCs/100,000 µm^2^), and it underwent a decrease of a similar order of magnitude in BU-fed salmons (93 ± 38 MMCs/100,000 µm^2^).

These preliminary observations could have important implications for the interpretation of the results of the RNA-sequencing experiment. Indeed, fish MMCs are cell aggregates mostly found in the fish head kidney and spleen, which are broadly recognized as histological indicators of the immune response [6]. MMCs consist of highly pigmented melanin-producing cells with phagocytic activity, i.e., melanomacrophages., which are thought to represent a phylogenetically relict population of CD83+ leukocytes, which carry out multiple immune and housekeeping functions [82]. Although MMCs are normally present in the hematopoietic tissues of healthy teleosts, their density can significantly increase in response to a wide range of stress conditions, including the exposure to pollutants and metabolic toxicants, extreme salt concentration, and starvation [83,84,85,86]. The melanin deposition process can in turn trigger an inflammatory response mediated by the deposition of the complement component C3 and by the activation of the alternative pathway [87].

While the increase in the density of head kidney MMCs in the KM group observed in this experiment seems to be consistent with the upregulation of C3 (Figure 1 and Table 1), the presence of comparable histological alterations in SM-fed salmons, where neither C3 nor other complement-related genes were differentially modulated, could not be ascribed to the activation of the classical pathway of the complement system. Although further investigations will be required to clarify this aspect, due to the low number of fish that have been examined, the increased melanization observed in SM-fed salmons may be linked to a generalized stress condition induced by this plant ingredient-based diet, which has been previously shown to induce enteritis in the Atlantic salmon intestinal tract [88,89].

The decreased density of MMCs in the BU group was counterintuitive, considering the fact that, as in KM, the C3 expression was markedly upregulated in response to butyrate (Figure 1 and Table 1). However, a possible explanation for this unexpected observation might be found in the contemporary upregulation of other negative regulators of the alternative pathway of the complement system, such as CFHL and CFHR5L (Table 1), which act either by inhibiting the activity of C3b and C5b convertases [50] or by accelerating their decay [52]. Hence, we propose the hypothesis, which needs to be confirmed in successive experiments, that the strong activation of C3 and CFBL in the BU group was not able to trigger the alternative pathway-mediated deposition of melanin due to the contrasting action of the upregulated complement inhibitory factors CFHL and CFHR5L, which resulted in an overall reduction of the MMC density compared with controls.

## 3. Conclusions

In summary, this long-term differential feeding experiment evidenced that just a low number of genes were significantly modulated in the head kidney of juvenile Atlantic salmon. Compared to the control, the most significant changes were observed in the BU-fed group (eight DEGs), followed by the KM-fed group (three DEGs), with partial overlap between the genes modulated by these two dietary supplementations. The SM-rich diet only produced nearly negligible modifications (one DEG), and the BA-rich diet did not result in a significant modulation of gene expression. Most of the 10 transcripts modulated by the diet were of immunological interest, and C3 and CFBL in particular were strictly linked with the function of the complement system, further supporting the strong involvement of this molecular machinery in the transcriptional response of farmed salmons to differential feeding, as previously reveled for the pyloric caeca [43]. The alteration of gene expression profiles was accompanied by interesting histological alterations in the head kidney, due to an increase in the density of MMCs in the KM and SM groups, and a decrease of the same parameter in the BU-fed group. Although further studies will be necessary, these preliminary histological data may be consistent with an increased activity of the alternative pathway of the complement system. While the results of this study suggest that butyrate-rich, and, in minor measures, also krill meal and soybean meal, feed may produce beneficial effects on juvenile farmed salmons, specifically linked with the immune stimulation of complement system activity, the functional significance of these alterations on salmon growth performance and health remain to be fully elucidated.

## 4. Methods

### 4.1. Experimental Setup

Atlantic salmon (*Salmo salar*) young individuals at the fry stage were obtained from a local hatchery (from Lerøy, mid As) and transferred to SeaLab aquaculture research facility (Department of Biology, NTNU, Trondheim, Norway). The fish were kept in a 400-L tank, 3 tanks per each feeding group, 30 fish per tank at the initial timepoint. The water system was flow through, and water flow was kept at 40 L/h, with a 24h/0h photoperiod (light/dark) and a stable temperature at 10 °C. In order to acclimate, all the fish were fed by commercial control feed using an automatic single feeder continuously in need and to excess, kept under standard temperature, salinity, and oxygen (over 80%) conditions, bulk weighed and randomly assigned to three replicate tanks. After 2 weeks of acclimation, animals were randomly divided in five feeding groups: Control (C), krill meal (KM), soybean meal (SM), Bactocell^®^ (BA), and butyrate (BU).

The feedings were provided as standard fish feed pellets by BioMar Co., Norway (C, KM, SM, and BU) and Lallemand Animal Nutrition, Canada (BA). The feed was supplied continuously and in excess using automatic feeders (Arvotec single feeder). All diets had the same ingredient composition, with the exception of the following compounds: The KM diet contained 7.4% Antarctic krill meal (*Euphausia superba*); the SM diet had 17.68% soya hp48 (soymeal, high protein hydrolyzed) and 5% soya SPC (refined soymeal); compared with SM, the BA diet contained *P. acidilactici* MA 18/5 M (Bactocell^®^), a live lactic acid bacterium that can convert complex carbohydrates to lactic acid; and the BU diet included 0.05% sodium butyrate 30% coated. The ingredients of the five feedings are detailed in Table 2.

At day 84, five specimens for each experimental group were sacrificed using 1 g/L of tricaine methanesolphonate (MS-222) (Sigma-Aldrich, Steinheim am Albuch, Germany). The head kidney tissues were dissected from the dorsal body wall of the fish with fine forceps. All samples were kept in 1 mL of RNA later^®^ solution (Sigma-Aldrich) for 24 h to ensure full permeation of the dissected tissues and samples were stored at −80 °C until RNA extraction.

### 4.2. RNA Extraction and RNA-Sequencing Analysis

The extraction of RNA was performed at the Fugelab, Department of Biology, NTNU, Norway. Thirty milligrams of head kidney tissue from four biological replicates were homogenized by TissueLyser (Qiagen, Hilden, Germany) for 120 s × 2,24 Hz. Total RNA was extracted with an Animal RNeasy mini kit (Qiagen) according to manufacturer’s instructions. Extraction was followed by an on-column DNase digestion using an RNase-free DNase (Qiagen) and spin RNA clean-up. The quantity of extracted RNA was measured by NanoDrop spectrophotometry (ND1000, v 3.7, Thermo Fisher Scientific, Waltham, MA, USA), revealing that all the selected samples displayed 280/260 and 260/230 ratios between 1.8 to 2.2. The quality of total RNA was determined with a Bioanalyzer 2100 (Agilent, Wilmington, DE, USA), revealing an RNA integrity (RIN) value higher than 9.5 for all samples. Sequencing libraries were prepared with a TruSeq Stranded mRNA Library Prep Kit (Illumina, San Diego, CA, USA) according to the manufacturer’s protocol. Libraries were sequenced on a HiSeq 2500 platform (Illumina) at the Norwegian Sequencing Facility (Oslo, Norway), using a single-end strategy with 120 cycles. Raw sequence data has been deposited in the NCBI SRA repository, under the BioProject PRJNA574264.

### 4.3. Gene Expression Analysis

Reads were quality assessed, trimmed to remove sequencing adapters and low-quality bases, and aligned to the HISAT2-indexed version of the *Salmo salar* genome [90] from salmobase.org using the HISAT2 software [91], with default parameters for single-end reads. Read counts in genomic regions annotated as transcribed were calculated with the feature Counts software [92], using Atlantic Salmon ICASG_v2 as the transcriptome reference. Read alignment and counting were performed within the Galaxy environment using a pre-designed workflow [93]. Read counts from mapping were converted in gene expression levels (which are reported in the present manuscript as transcripts per million (TPM)) [94]. Digital gene expression data were analyzed through the Voom package [95,96] by comparing the transcriptional profiles of the KM, SM, BA, and BU-fed with the C group. DEGs were detected based on the following thresholds: False discovery rate (FDR) adjusted *p*-value < 0.05; FC > |2|.

### 4.4. Validation of Gene Expression Trends by RT-qPCR

Complementary DNA (cDNA) was synthesized with a QuantiTect Reverse Transcription Kit (Qiagen, Hilden, Germany) using 1µg of total RNA previously extracted from each fish as a template, according to the manufacturer’s instructions. The cDNA was diluted 10 times before reverse transcriptase-quantitative polymerase chain reaction (RT-qPCR) assay. The amplification reactions were performed on a Lightcycler^®^ 96 instrument, using a Lightcycler 480 SYBR-green I Master kit (Roche Applied Science, Germany). All PCR reactions were carried out with three technical replicates. The PCR reaction program was set as follows: 1 min initial denaturation at 95 °C; 40 amplification cycles consisting of 10 s denaturation at 95 °C, 10 s annealing at 55 °C, and 10s elongation at 72 °C. The specific amplification of the target genes was assessed with a melting curve analysis.

In total, 9 out of 10 genes showing statistically significant differential expression by in silico analysis were selected to validate RNA sequencing data by RT-qPCR assay. The selected genes were VTG, MFAP4, CFHL, TM4SF4, CBLN2L, APOBL, CFHR5L, CFBL, andC3. CDH4 was not selected due to the very low expression levels observed by RNA-seq analysis. The elongation factor-1AA and -1AB were used as housekeeping genes, since they have been previously shown as proper internal controls for normalization in Atlantic salmon [97,98]. Primers are provided in Table 3. The efficiency of PCR reactions was calculated using the LinRegPCR gene quantification software (Heart Failure Research Center, Netherlands), and mean Ct values were used to calculate statistical significance differences in the gene expression levels for comparison between two study groups (KM, SM, BA, and BU vs C) with the Qbase+ software (Biogazelle Co., Gent, Belgium). The significance of the correlation between RNA-seq and PCR-based expression values was tested with the Pearson correlation coefficient, excluding two outliers (CFHR5L and TM4SF4).

### 4.5. Histological Image Analysis

Two fish from each feeding group were randomly collected at the end of the experiment and the head kidney tissue from the dorsal body wall was dissected on chilled trays. The samples were kept in paraformaldehyde 1% to be chemically fixated, dehydrated, and subsequently transferred to ethanol 70% and embedded in the proper cassettes until further processing. After fine sectioning, multiple sets of sections were stained by hematoxylin and eosin.

Cell measurements were obtained using a computer-assisted image analysis system, which included a Zeiss microscope equipped with a color 8 video camera (Axio Cam MRC) and a software package (KS 300 and AxioVision). The density of MMCs was calculated as the mean + SEM in a pool of two specimens for each experimental group.

### 4.6. Availability of Data and Materials

All the raw sequencing data has been deposited in the NCBI SRA repository, under the BioProject PRJNA574264.

### 4.7. Ethics Approval

The animal trial on juvenile Atlantic salmon specimens has been approved by the Animal Welfare Committee, Norwegian University of Science and Technology (NTNU) and all transfer, breeding, anesthesia, and killing procedures have been conducted taking into consideration Norwegian Food Safety Authority (Mattilsynet) regulations at SeaLab Animal Lab (NTNU) (FOTS ID:9067). The quality of water, salinity, temperature, noise and catching procedures have been checked carefully with respect to regulations for the use of live animals during the experiments. The ethical justification for endpoints and euthanasia were considered regarding the latest available guidance.

## Figures and Tables

**Table 1:** Summary of DEGs in the head kidney of the five different groups after 12 weeks. ns = not significant.

NCBI Gene ID	Gene Acronym	KM vs C		SM vs C		BA vs C		BU vs C	
		LogFC	FDR	LogFC	FDR	LogFC	FDR	LogFC	FDR
100136426	VTG	ns	ns	1.56	3.95 × 10^−2^	ns	ns	2.83	9.74 × 10^−6^
100196216	MFAP4	3.76	1.66 × 10^−2^	ns	ns	ns	ns	ns	ns
106560553	CFHL	ns	ns	ns	ns	ns	ns	2.15	1.52 × 10^−2^
106560595	TM4SF4	ns	ns	ns	ns	ns	ns	1.7	1.22 × 10^−2^
106566735	CDH4	ns	ns	ns	ns	ns	ns	4.17	3.03 × 10^−2^
106571136	CLBLN2L	2.81	2.82 × 10^−3^	ns	ns	ns	ns	ns	ns
106571178	APOBL	ns	ns	ns	ns	ns	ns	1.99	5.52 × 10^−4^
106598656	CFHR5L	ns	ns	ns	ns	ns	ns	1.8	1.34 × 10^−2^
106602980	CFBL	ns	ns	ns	ns	ns	ns	1.81	1.34 × 10^−2^
106604987	C3	1.73	5.04 × 10^−3^	ns	ns	ns	ns	2.08	4.08 × 10^−4^

VTG: vitellogenin; MFAP4: microfibrillar-associated protein 4; CFHL: complement factor H-like; CFBL: complement factor B-like; APOBL: apolipoprotein B-100-like; C3: complement 3; CBLN2L: cerebellin-2-like protein; CFHR5L: complement factor H-related protein 5-like; TM4SF4: transmembrane 4L6 family member 4-like.

**Table 2:** Composition (%) of the five feeds used in the present study. Control diet ©, krill meal (KM), soybean meal (SM), Bactocell^®^ (BA), and butyrate-rich (BU) diets.

Composition	C	KM	SM	BA	BU
Fish Meal LT	12.45	10.1	14.16	14.14	14.12
Fish Meal SA Superprime	12.5	7.5	11.84	11.86	11.88
Krill meal		7.4			
Soya HP48, Non-GMO			17.68	17.64	17.61
Soya SPC, Non-GMO	18.05	18	5	5	5
Wheat Gluten,	7.7	10	14.1	14.1	14.1
Maize Gluten 60	5	5			
Pea Protein	10	7.58	5	5	5
Wheat	12.5	12.5	11.2	11.2	11.2
Fish Oil, 18 EPA+DHA	9.71	8.71	9.82	9.82	9.82
Rapeseed Oil, Crude	9.44	9.82	9.91	9.91	9.91
Vitamin and minerals	2.14	2.41	2.00	2.00	**2.00**
AA-mix	0.82	1.04	0.97	0.97	**0.97**
Bactocell PA10				0.03	
Sodium Polyhydroxy Butyrate 30% coated					**0.05**
YTTRIUM	0.05	0.05	0.05	0.05	0.05
Lucantin Pink CWD 10%	0.05	0.05	0.05	0.05	0.05
Water change	−0.4	−0.15	−1.76	−1.76	−1.75
**Total**	**100**	**100**	**100**	**100**	**100**

**Table 3:** List of the primers used in this study.

NCBI Gene ID	Gene Acronym	Forward Primer (5′-> 3′)	Reverse Primer (5′-> 3′)
100136426	VTG	GTCTCTCTATGCCCCAAGCC	TCCACAGGTCTGTCCCTTCA
100196216	MFAP4	GGCTAAAGTCCACGTCCAGT	CCGGCACCTCCATCTTTGAA
106560553	CFHL	ATGCCCAGTGATTCAAGCCA	CAGTAGCTACAGTTTACCTTCACA
106560595	TM4SF4	CCATCCAGGTCATCAACGGT	AGCAAAAGGCCGTCAAGCTA
106571136	CLBLN2L	TTGGGAATTCAGGGAAGGCG	CCGGATTTTGGGTTTGCAGT
106571178	APOBL	TCCCCAGAAGATAGCCGACA	TGCAATGTTTTCTGCAGCCC
106598656	CFHR5L	TTGCCAATCTGGAGGATGGA	GACGACCCCAGTAATCCTTTTG
106602980	CFBL	AGAGGGAATCACCTGCAAGC	ACAGATTTACGGTGCCCCAG
106604987	C3	TCGATTTGGTCGTCAAGCCA	GCAGGTCTTCAGACTTCCCC

## Abbreviations

APOBL	apolipoprotein B-100-like
BA	Bactocell^®^
BU	butyrate
C	control
C3	complement component C3
CFBL	complement factor B-like
CBLN2L	cerebellin 2-like
CFHR5L	complement factor H-related protein 5-like
CDH4	cadherin 4
CFHL	complement factor H-like
DEGs	differentially expressed genes
FC	fold change
FHR	factor H-related proteins
FDR	false discovery rate
HISAT2	hierarchical indexing for spliced alignment of transcripts
KM	krill meal
MFAP-4	microfibril-associated protein 4
MMCs	melanomacrophage centers
RT-qPCR	reverse transcriptase- quantitative polymerase chain reaction
SM	soybean meal
TM4SF4	transmembrane 4L6 family member 4-like
TPM	Transcripts Per Million
VTG	vitellogenin
